# ﻿Description of the first stygobiotic species of the atyid shrimp genus *Sinodina* (Decapoda, Caridea, Atyidae) from Yunnan Province, China

**DOI:** 10.3897/zookeys.1186.112657

**Published:** 2023-12-12

**Authors:** Xuankong Jiang, Jiajun Zhou, Jianguo Wang, Wenlong Chen, Huiming Chen

**Affiliations:** 1 Guizhou Institute of Biology, Guizhou Academy of Sciences, 1 Longjiang Lane, Guiyang 550009, Guizhou, China Guizhou Institute of Biology, Guizhou Academy of Sciences Guiyang China; 2 Zhejiang Forest Resource Monitoring Center, Hangzhou 310020, China Zhejiang Forest Resource Monitoring Center Hangzhou China; 3 Zhejiang Forestry Survey Planning and Design Company Limited, Hangzhou 310020, China Zhejiang Forestry Survey Planning and Design Company Limited Hangzhou China; 4 Pearl River Water Resources Research Institute, Pearl River Water Resources Commission of the Ministry of Water Resources, Guangzhou,510610, China Pearl River Water Resources Research Institute, Pearl River Water Resources Commission of the Ministry of Water Resources Guangzhou China; 5 Key Laboratory of the Pearl River Estuary Regulation and Protection of Ministry of Water Resources, Guangzhou 510611, China Key Laboratory of the Pearl River Estuary Regulation and Protection of Ministry of Water Resources Guangzhou China

**Keywords:** Diversity, morphology, new species, phylogeny, stygobiont, taxonomy

## Abstract

​*Sinodina* Liang & Cai, 1999, a genus of atyid shrimp, is endemic to China and distributed only in the Yunnan-Guizhou Plateau. We describe here the thirteen species of *Sinodina*, and the first cave-dweller of the genus, *Sinodinaashima***sp. nov.**, collected from a limestone cave in Shilin County, Yunnan Province. This species can be distinguished from its congeners by the completely degraded pigment and eyes, the extremely long rostrum, the rostral formula and the absence of sexual dimorphism of the third and fourth pereiopods. A phylogenetic analysis based on four genes (COI, 16S, 18S, H3) shows that the new species strongly clustered with the type species of this genus, *Sinodinagregoriana* (Kemp, 1923), supporting the generic status of this new species.

## ﻿Introduction

The genus *Sinodina* Liang & Cai, 1999 belongs to the order Decapoda and the family Atyidae. It was established by Liang and Cai (1999) on the type species *Caridinagregoriana* Kemp, 1923. In the paper, the authors also transferred *Caridinayui* Liang & Yan, 1985, *Caridinaacutipoda* Liang, 1989 and *Caridinabispinosa* Liang & Yan, 1990 (in Liang 1990) to *Sinodina*, and published three new species, *Sinodinadianica* Liang & Cai, 1999, *S.wangtai* Liang & Cai, 1999 and *S.lijiang* Liang & Cai, 1999. Chen and Liang (2002) described a new species *Sinodinayongshengica* Chen & Liang, 2002 from Yongsheng, Yunnan, China. Liang (2002) based on the specimens from Jiangchuan, Yunnan, described *Sinodinaangulata* Liang, 2002. Liang (2004) reviewed the genus and placed *Caridinaleptopropoda* Liang, 1990, *Caridinaheterodactyla* Liang & Yan, 1985 and *Caridinabanna* Cai & Dai, 1999 into *Sinodina*. Ultimately, a total of 12 species have been recorded (Liang 2004; De Grave and Fransen 2011), making it the third largest genus of Atyidae in the Chinese fauna, after *Caridina* and *Neocaridina*. All of these species are endemic to a narrow range and only distributed in Yunnan Province, southwest China, except *Sinodinagregoriana* (Kemp, 1923), which has a relatively larger distribution range, not only in some lakes in Yunnan but also in Caohai Lake, Guizhou Province (Liang 2004).

The morphology of *Sinodina* is similar to that of the genera *Caridina* and *Neocaridina*, and they share the same branchial formula. *Sinodina* can be identified by the simple and lamellar podobranch of the second maxilliped and the obvious sexual dimorphism, that is, the male possesses more spines and distinctive dilation on the propodus of the third and fourth pereiopod (Liang and Cai 1999). According to Liang (2004), both the simple podobranch and the dilated pereiopod with a large number of spines of the male are plesiomorphic. Thus, *Sinodina* probably is a more basal group than *Caridina* and *Neocaridina*.

There are numerous caves in the karst areas of south China, which provided refuge for organisms in this area during the Neogene when the climate and habitat had been changing, especially after the Oligocene-Miocene boundary (Li et al. 2022). Many species have adapted to the cave environment and have undergone morphological changes, such as degeneration of eye and body coloration and elongation of the limbs. Some of them have completely adapted to the subterranean surroundings and live exclusively in the cave, becoming stygobionts/troglobionts. Cave shrimp is an interesting group among the stygofauna of this region. At present, four genera (*Caridina*, *Mancicaris*, *Neocaridina* and *Typhlocaridina*) and 27 species of Atyidae have been discovered and described from Chinese caves, distributed in Guangxi (12 species), Guizhou (8 species), Yunnan (3 species), Hunan (3 species) and Hubei (1 species) provinces (Cai and Ng 2018; Xu et al. 2020; Feng et al. 2021; Guo et al. 2022), and the number has continued to increase.

We surveyed Xiangshuiqing Cave in Shilin County, Yunnan Province twice in April and June 2023 and collected a total of 14 atyid shrimp specimens with strong cave morphological features. They were identified as a new species of *Sinodina* through morphological observations and molecular analysis. This species is the first stygobiont in the genus and the fourth cave atyid species in Yunnan Province, after three *Caridina* species, *Caridinafeixiana* Cai & Liang, 1999, *Caridinaalu* Cai & Ng, 2018 and Caridinaaff.heterodactyla Liang & Yan, 1985 (Cai and Ng 2018).

## ﻿Materials and methods

### ﻿Specimen collecting and preservation

Specimens were collected by cage nets from a limestone cave in Shilin, Yunnan, southern China. Live animals were observed and photographed with a Sony A7R4A camera with a Sony FE 90 mm macro lens. Most of the specimens were preserved in 75% ethanol for morphological studies, and the remainder were preserved in absolute ethanol and stored at –40 °C for molecular research. All specimens are deposited at the Institute of Biology, Guizhou Academy of Sciences, Guiyang, China (IBGAS).

### ﻿Morphological study

Specimens were examined, photographed and measured using a Leica M205A stereomicroscope equipped with a Leica DFC450 camera and LAS X software (v. 5.1, Leica, Germany). All images were edited with PHOTOSHOP CC 2019 software (v. 20.0.0, Adobe, USA).

The following abbreviations are used in the text: **alt** (altitude), cl (carapace length, measured from the postorbital margin to the posterior margin of the carapace), **rl** (rostral length, measured from the rostral tip to the postorbital margin) and tl (total length, measured from the rostral tip to the posterior margin of the telson). All measurements are in millimeters.

### ﻿Molecular analyses

To verify the classification of the new species, a multi-genes phylogenetic analysis was conducted. Four specimens of *Sinodinaashima* sp. nov. were sampled. The ingroup of the matrix was composed of *Sinodinagregoriana* (Kemp, 1923), two cave-dweller species of *Caridina*, *Caridinacavernicola* Liang & Zhou, 1993 and *Caridinasinanensis*Xu et al., 2020, two species of *Neocaridina*, *Neocaridinapalmata* (Shen, 1948) and *Neocaridinahofendopoda* (Shen, 1948), and *Paracaridinaguizhouensis* (Liang & Yan, 1986). *Macrobrachiumnipponense* (De Haan, 1849) of Palaemonidae was selected as the outgroup. Detailed geographical information and sequence metadata are listed in Table 1.

**Table 1.** Details of the specimens used for the molecular analyses.

Taxon	Voucher number	Collection data	GenBank number				Reference
			COI	16S	18S	H3	
*Sinodinaashima* sp. nov.	GBZD-676	Xiaoliao Cave, Shilin, Yunnan, China, 4. VI. 2023, X.K. Jiang leg.	–	OR537884	OR539523	–	This study
	GBZD-677		OR536642	OR537885	OR539524	–	This study
	GBZD-678		OR536643	OR537886	OR539525	–	This study
	GBZD-679		OR536644	OR537887	OR539526	–	This study
*Sinodinagregoriana*	GBZD-238	Yangwanqiao Reservoir, Weining, Guizhou, China, 17. X. 2020, X.K. Jiang & H.M. Chen leg.	–	OR537881	OR539518	OR540202	This study
	GBZD-239		–	–	OR539519	OR540203	This study
	GBZD-240		–	–	OR539520	OR540204	This study
	GBZD-241		–	OR537882	OR539521	OR540205	This study
*Sinodina* sp.	ZMB DNA-651	Yunnan, China	–	FN995388	–	–	von Rintelen et al. 2012
*Caridinacavernicola*	–	Hechi, Guangxi.	MZ753498	MZ753801	–	–	Guo et al. 2022
*Caridinasinanensis*	–	Sinan, Guizhou, 25. I. 2019	MT433963	MT434874	–	–	Xu et al. 2020
*Neocaridinapalmata*	GBZD-098	Lisong, Hezhou, Guangxi, China, 25. IV. 2021, X.K. Jiang leg.	OR536639	–	OR539516	OR540200	This study
*Neocaridinahofendopoda*	GBZD-141	Sijia River, Yacai, Sanjiang, Guangxi, China, 15. III. 2021, X.K. Jiang, H.M. Chen & J.C. Lv leg.	OR536640	–	OR539517	OR540201	This study
*Paracaridinaguizhouensis*	GBZD-562	Longquan, Maopo, Yuping, Guizhou, China, 29. IV. 2022, X.K. Jiang, H.M. Chen & L.P. Ye leg.	OR536641	OR537883	OR539522	OR540206	This study
*Macrobrachiumnipponense*	GBZD-001	Guangzhao Reservoir, Qinglong, Guizhou, China, 14. I. 2021, H.M. Chen leg.	OR536638	OR537880	OR539515	OR540199	This study

Four loci, including two mitochondrial genes (cytochrome c oxidase subunit I and 16S rDNA) and two nuclear genes (18S rDNA and histone H3 gene) were used to conduct the analysis. Primer sequences are in Table 2. Except for that of *Caridinacavernicola* and *Caridinasinanensis*, all sequences of this matrix were obtained in this research.

**Table 2.** Primers used for PCR and sequencing.

Genes	Primer	Sequence (from 5’ to 3’)	Reference
COI	LCO1490	GGTCAACAAATCATAAAGATATTGG	Folmer et al. 1994
	HCO2198	TAAACTTCAGGGTGACCAAAAAATCA	
16S	16sA	ACTTGATATATAATTAAAGGGCCG	Wowor et al. 2009
	16sB	CTGGCGCCGGTCTGAACTCAAATC	
18S	18s ai	CCTGAGAAACGGCTACCACATC	DeSalle et al. 1992
	18s bi	GAGTCTCGTTCGTTATCGGA	
H3	H3 AF	ATGGCTCGTACCAAGCAGAC(AGC)GC	Colgan et al. 1998
	H3 AR	ATATCCTTRGGCATRARTGTGAC	

Raw sequences were edited and assembled using SEQMAN PRO software (Lasergene v. 7.1; DNA Star, Inc., Madison, Wis., USA). Protein-coding gene sequences (COI and H3) were aligned based on amino acid translation using CLUSTALW in MEGA 7.0 (Kumar et al. 2016). The more variable sequences (16S and 18S) were aligned using the online version of MAFFT v. 7.0 (Katoh and Standley 2013) under the algorithm, Q–INS–i. All other settings were left as default. After manual trimming, the resulting sequences were concatenated using MESQUITE v. 3.6 (Maddison and Maddison 2015).

PARTITIONFINDER 2 (Lanfear et al. 2016) was used to determine the optimum partitioning scheme and the best-fitting model for each partition, using the corrected Akaike Information Criterion (AICc). We input the partition file that contained six partitions, in which the protein-coding genes (COI and H3) was divided into codon positions for each fragment.

Maximum likelihood (ML) and Bayesian inference (BI) analyses were conducted to infer the phylogeny. ML was performed in RAXML v. 8.2.0 (Stamatakis 2014) under a GTRGAMMA model, and the six partitioning schemes, using 1000 rapid bootstrap replicates and a random seed value set to 12345. BI was implemented in MRBAYES v. 3.2.5 (Ronquist et al. 2012) following the parameters obtained from PARTITIONFINDER and with two simultaneous Monte Carlo Markov (MCMC) runs for 1 million generations, and tree samples were output every 1000 generations with a burn-in of 25%. Trees were visualized and edited with FIGTREE v. 1.44 (Rambaut 2016).

In addition, the pairwise p-distances between COI and 16S genes of all specimens of *Sinodinaashima* sp. nov. and *Sinodinagregoriana* were calculated with MEGA 7.0. One 16S sequence of *Sinodina* sp. derived from Genbank (Table 1) was also calculated for their interspecific distances.

## ﻿Results

### ﻿Taxonomy

#### ﻿ Sinodina ashima sp. nov.

Taxon classificationAnimaliaDecapodaAtyidae

DD90F4FB-F4A0-51F4-ABCA-6C41FE1F3718

https://zoobank.org/B4D759DE-18AD-4F4E-A47D-16DE20D99B13

**Figure 1.**
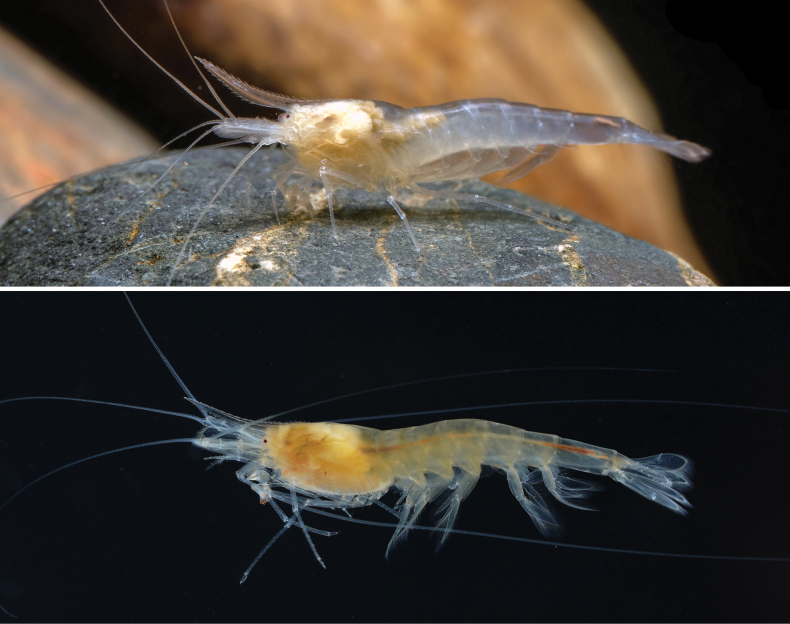
Live specimens of *Sinodinaashima* sp. nov.

**Figure 2.**
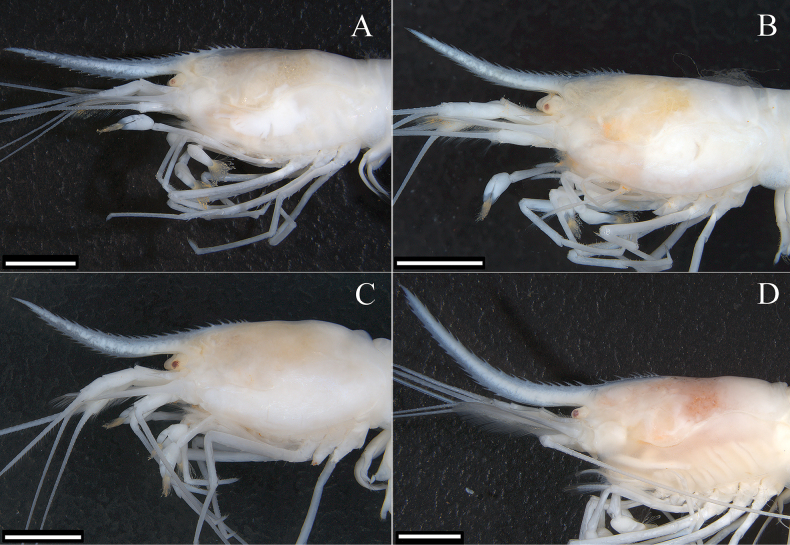
Cephalothorax of *Sinodinaashima* sp. nov., lateral view, showing the variation of the rostrum **A** female paratype, tl 23.5 mm **B** holotype **C** male paratype, tl 27.5 mm **D** female paratype, tl 40 mm. Scale bars: 2.5 mm.

**Figure 3.**
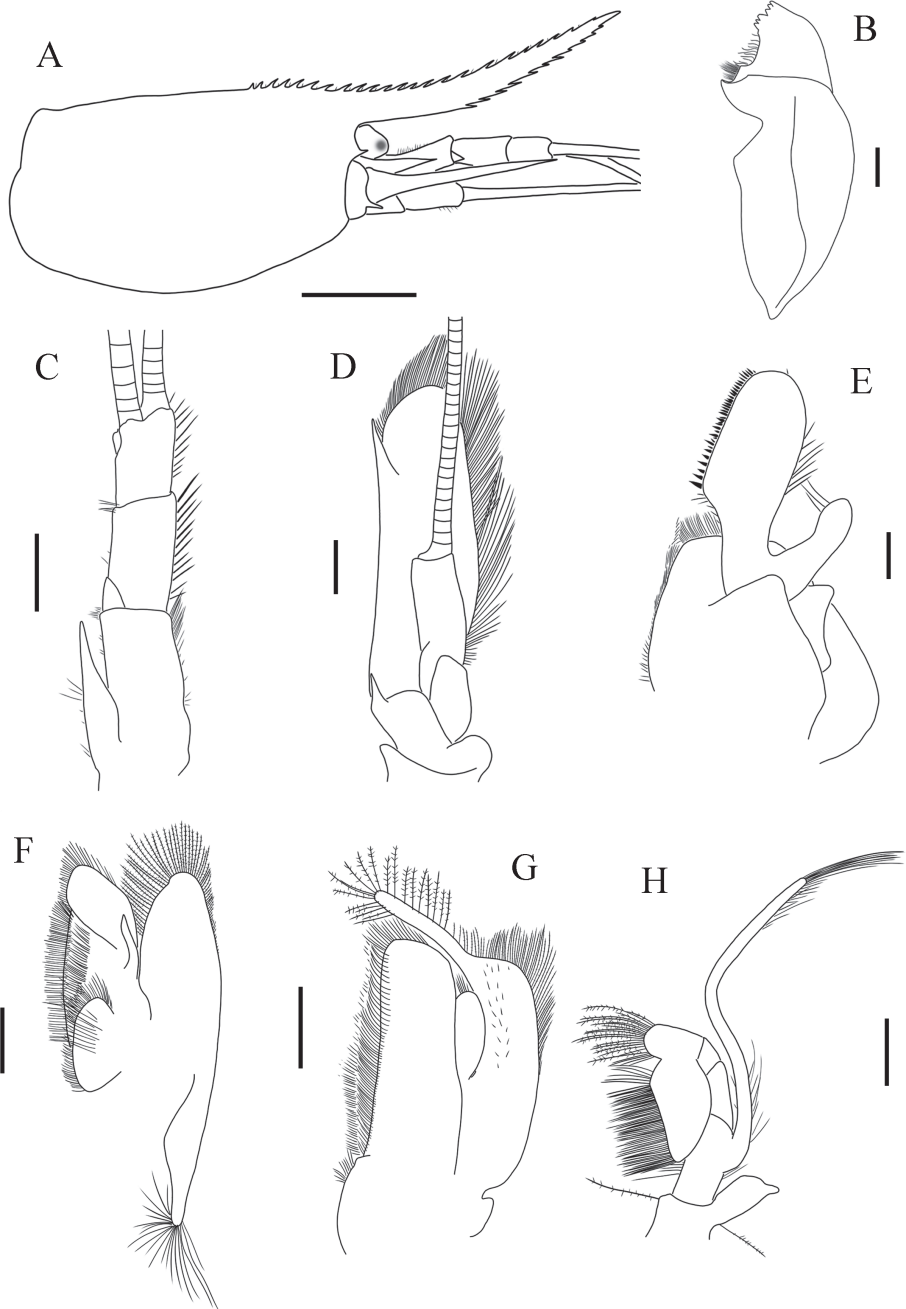
Holotype of *Sinodinaashima* sp. nov. **A** cephalothorax and cephalic appendages, lateral view **B** mandible **C** antennule **D** antenna **E** maxillula **F** maxilla **G** first maxilliped **H** second maxilliped. Scale bars: 2.5 mm (**A**); 0.25 mm (**B, E**); 0.75 mm (**C, D**); 0.5 mm (**F–H**).

**Figure 4.**
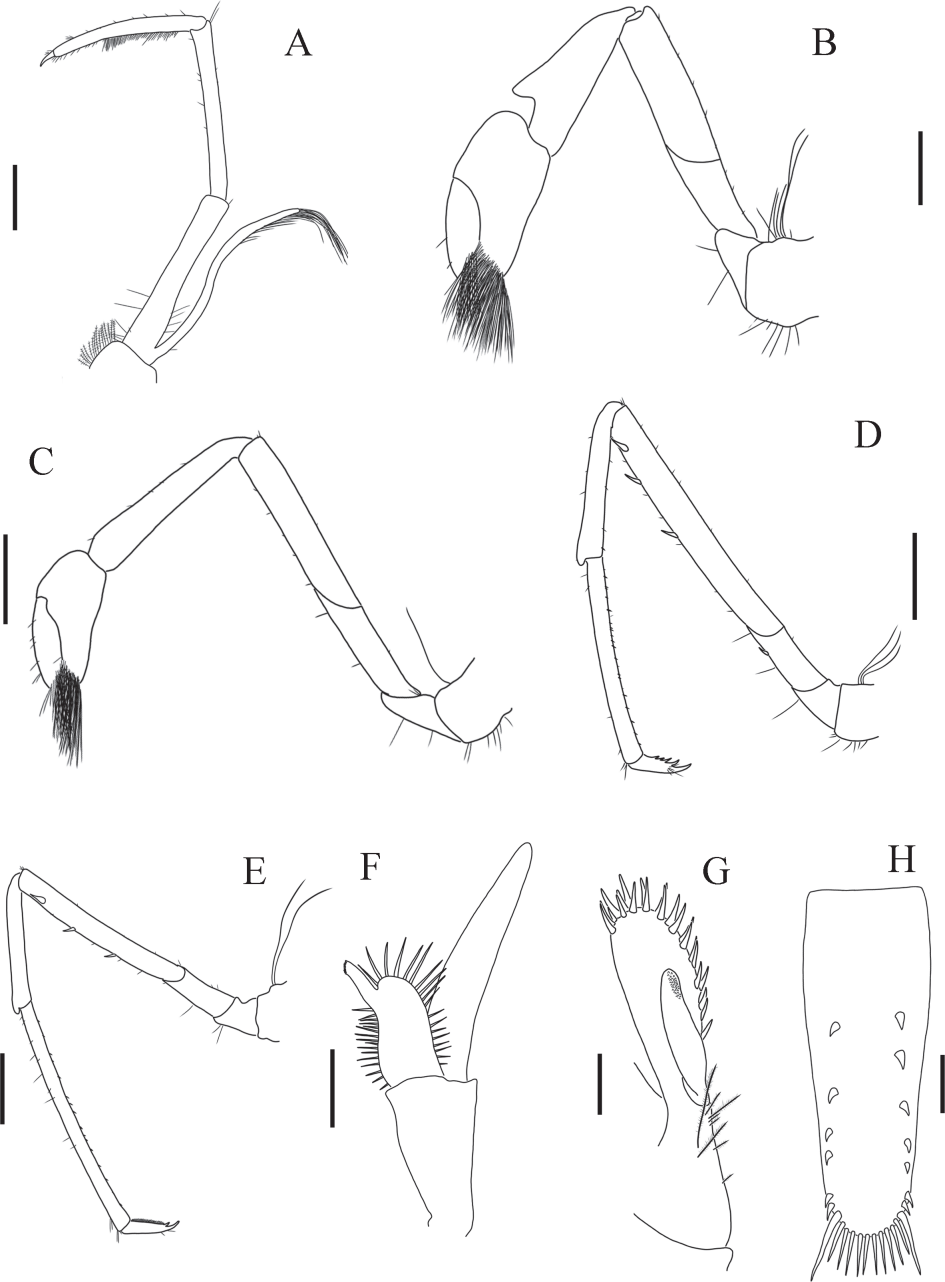
Holotype of *Sinodinaashima* sp. nov. **A** third maxilliped **B** first pereiopod **C** second pereiopod **D** third pereiopod **E** fifth pereiopod **F** first pleopod **G** appendix masculina and appendix interna of second pleopod **H** telson. Scale bars: 0.75 mm (**A, C, F**); 0.5 mm (**B, H**); 1 mm (**D, E**); 0.25 mm (**G**).

**Figure 5.**
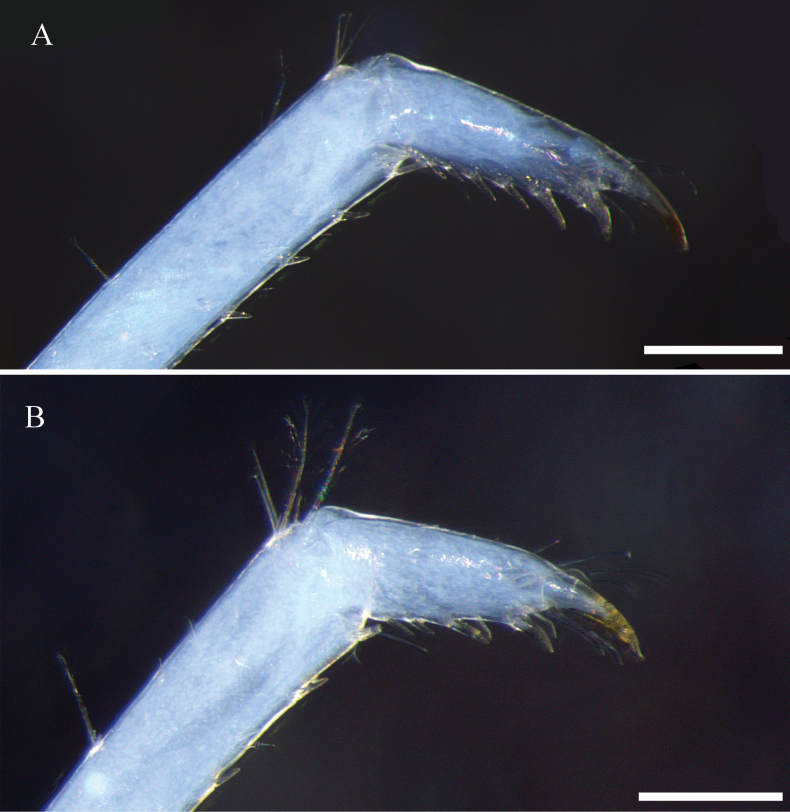
Dactylus of third pereiopod **A** holotype **B** female paratype. Scale bars: 0.25 mm.

Figs 1 , 2 , 3 , 4 , 5

##### Type material.

***Holotype***: male (rl 5.1 mm, cl 5.8 mm, tl 26.7 mm), China, Yunnan Province, Kunming City, Shilin County, Xiangshuiqing Cave, 24°45′27.53″N, 103°19′54.88″E, alt. 1790 m, 4. VI. 2023, Jiang X.K. leg. ***Paratypes*.** 2 males (rl 5.0–5.9 mm, cl 6.0–6.6 mm, tl 27.5–29.5 mm) and 8 females (rl 4.6–6.5 mm, cl 5.4–6.6 mm, tl 23.5–28.7 mm), collected with holotype; 3 females (rl 5.7–9.0 mm, cl 6.7–8.2 mm, tl 28.9–40.0 mm), same locality, III. 2023, Zhou J.J. leg.

##### Diagnosis.

Body color and eyes strongly degenerated. Rostrum extremely elongated and upturned, obviously beyond end of scaphocerite, rostral formula: 7–11 + 14–15/8–14. Male propodus of third and fourth pereiopod normal without dilation. Dactylus of third pereiopod with 4–6 spinules. Telson with 6–7 pairs of dorsal spines.

##### Description.

***Body slender*** (Fig. 1). Rostrum long, slightly to strongly upturned (Fig. 2), reaching obviously beyond end of scaphocerite, 0.85–1.1 times of cl, armed dorsally with 22–26 (holotype 23) teeth, including 7–11 (holotype 8) situated posterior to orbital margin, ventrally with 8–14 (holotype 11) teeth, rostral formula: 7–11 + 14–15/8–14 (Figs 1–3A).

***Eyes*** small, highly reduced, without ocular peduncle, only centre of cornea slightly pigmented (Figs 1–3A).

***Carapace*** smooth, glabrous, antennal spine acute, pterygostomian margin subrectangular, pterygostomian spine absent (Figs 1–3A).

***Antennule*** (Fig. 3C) peduncle three-segmented, c. 0.6 times as long as carapace. Basal segment about 1.5 times as long as second and 2.0 times as long as third. All segments with submarginal setae. Stylocerite almost reaching end of basal segment. Anterolateral angle reaching one third of 2^nd^ segment. Flagella long and simple.

***Antennal*** (Fig. 3D) peduncle about 0.4 length of scaphocerite. Scaphocerite about 3.0 times as long as wide, outer margin straight, asetose, ending in a strong sub-apical spine, inner and anterior margins with long plumose setae.

***Mandible*** incisor process with six irregular and blunt teeth. Molar process truncated (Fig. 3B).

***Maxillula*** (Fig. 3E) lower lacinia broadly rounded, with several rows of plumose setae. Upper lacinia elongate, with numerous small teeth and short setae on inner margin. Palp digitiform, slightly expanded distally, with few long setae.

***Maxilla*** (Fig. 3F) with palp slender and slightly curved. Upper endites subdivided. Scaphognathite tapering posteriorly with some long, curved setae.

***First maxilliped*** (Fig. 3G) epipod small. Palp rounded, with several terminal plumose setae. Exopod flagellum distinct, well developed and with plumose marginal setae. Caridean lobe narrow, with dense plumose marginal setae.

***Second maxilliped*** (Fig. 3H) slender. Ultimate and penultimate segments of endopod fused. Inner margin of ultimate, penultimate and basal segments with long straight setae. Exopod long and slender, with several plumose setae distally. Podobranch simple.

***Third maxilliped*** (Fig. 4A) endopod three-segmented, basal segment about 7 times as long as broad, second segment about 10 times as long as broad and 0.95 times as long as basal segment, distal segment as long as second segment, ending in small claw-like apical spine surrounded by simple setae, preceded by 7 spines along distal third of posterior margin, a clump of long and simple setae proximally. Exopod reaching beyond end of basal segment of endopod, with long plumose setae distally.

***First pereiopod*** (Fig. 4B) stout, chela about 1.8 times as long as wide, 0.9 times length of carpus, movable finger about 2.8 times as long as wide, and 1.2 times length of palm, fingertips rounded, with numerous long setae. Carpus excavated anterodorsally, 2.3 times as long as wide and as long as merus. Merus slightly narrower than carpus. Ischium about 0.5 length of merus and about 2 times as long as basis.

***Second pereiopod*** (Fig. 4C) slender and longer than first pereiopod. Chela 2.2 times as long as wide, 0.72 times length of carpus. Movable finger 3.5 times as long as wide and 1.5 times as long as palm, setal brushes well developed. Carpus 5.2 times as long as wide, distal part normal, about 0.7 times length of merus.

***Third pereiopod*** (Fig. 4D) slender. Dactylus 2.8 times as long as wide (Fig. 5A) (female 2.4, Fig. 5B), ending in prominent claw-like spine surrounded by simple setae and 4–6 spines. Propodus 5.5 times as long as dactylus, bearing about 20 thin spinules evenly and loosely distributed on ventral margin, 13.5 times as long as wide. Carpus 0.71 times length of propodus. Merus 1.8 times length of carpus, with about 3–4 strong spines on the posterior margin. Ischium with a spine on the posterior margin.

***Fifth pereiopod*** (Fig. 4E) dactylus 3.6 times as long as wide, ending in prominent claw-like spine surrounded by simple setae, inner margin with about 30 and comb-like spines. Propodus 5.7 times length of dactylus, bearing about 15 spinules in two rows on ventral margin, 19.4 times as long as wide. Carpus 0.51 times length of propodus. Merus 1.5 times length of carpus, with about 3 strong spines on the posterior margin. Ischium about 0.3 times length of merus and 2.1 times length of basis.

***First pleopod*** (Fig. 4F) endopod tongue-like, about 2.0 times as long as wide, 0.4 times length of exopod, both inner and outer margin with spine setae, appendix interna well developed, arising from distal 1/5 of endopod, overreaching end of endopod, with cincinuli distally. Exopod 5.3 times as long as wide.

***Second pleopod*** endopod slender. Appendix masculina (Fig. 4G) strong, about 3/5 length of endopod, bearing about 25 long, spine-like setae distally as well as on distal part of inner margin. Appendix interna of endopod reaching 1/2 of appendix masculina, with cincinuli distally (Fig. 4G).

***Telson*** (Fig. 4H) about 0.5 times the postorbital carapace length and as long as sixth abdominal somite, tapering posteriorly and ending in a small median projection, dorsal surface with about 6–7 pairs of submarginal spines. Posterior margin with a pair of outermost spines and 5 pairs of intermediate spines that are slightly shorter than the lateral pair. Exopod of uropod longer and wider than endopod, both with plumose marginal setae. Diaeresis bearing 8–11 (holotype 11) spines.

***Eggs*** 0.85–0.91 × 1.20–1.27 in diameter.

***Color*** strongly degenerated, translucent to flavescent (Fig. 1).

##### Etymology.

The specific name is in honor of Ashima, who is a famous female character of the local legend spreading among the Yi nationality and is a symbol of love and bravery.

##### Distribution.

Yunnan Province (Xiangshuiqing Cave), China.

##### Habitat.

Subterranean river in a karst cave.

### ﻿Molecular analyses results

The phylogenetic matrix included 14 terminals with 2262 nucleotides (COI: 647 bp; 16S: 453 bp; 18S: 867 bp; H3: 295 bp). The best-fitting evolutionary model for the first codon of COI, 18S and the first and second codons of H3 was TRNEF+I+G. The best model for the second codon of COI was HKY+G. TRNEF+I was the optimal model for the third codon of COI. HKY+I+G and TVM+G suited the 16S and the third codon of H3 respectively.

**Figure 6.**
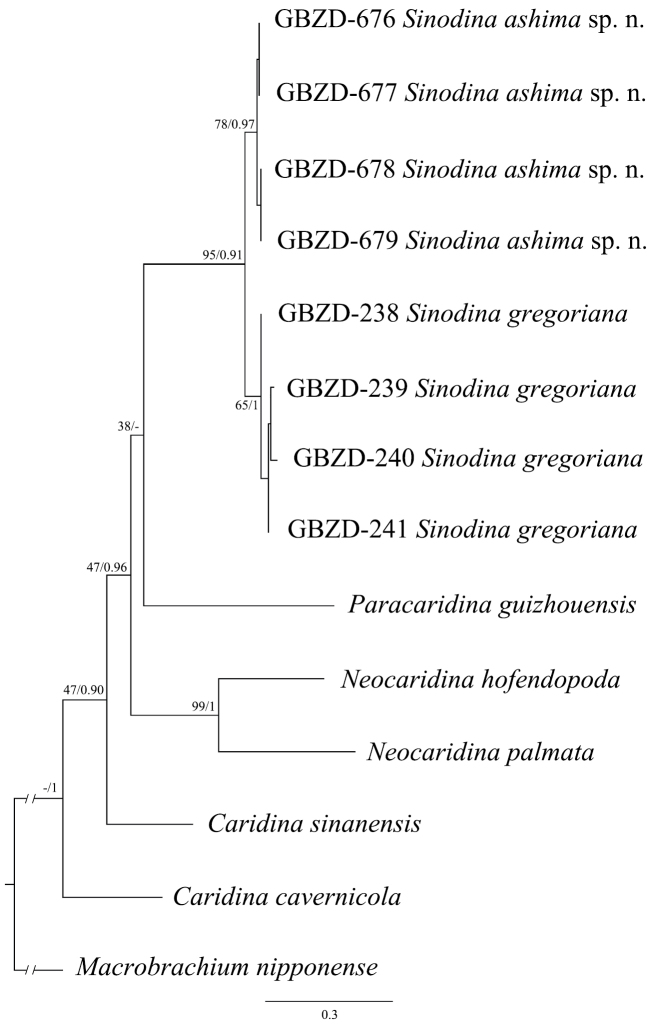
ML tree based on the concatenated dataset (COI + 16S + 18S + H3). Numbers at nodes are maximum likelihood percent bootstrap values (left) and Bayesian posterior probabilities (right).

The only difference between the topologies derived from the ML and BI analyses was the position of *Paracaridinaguizhouensis*. It was either sister to *Sinodina* spp. (ML) (Fig. 6) or clustered with the clade of *Sinodina* spp. and *Neocaridina* spp. (BI). Two pairs of sister species received strong support. One clade showed that the new species, *Sinodinaashima* sp. nov., was clustered with *Sinodinagregoriana* (bootstrap value and posterior probability = 95% and 0.91). Another branch contained the two *Neocaridina* spp. (bootstrap value and posterior probability = 99% and 1).

The COI sequences were successfully obtained from three specimens of *Sinodinaashima* sp. nov., but failed in all specimens of *Sinodinagregoriana*. The intraspecific p-distances of COI of the new species were 0% and 1.85%. Nevertheless, the 16S sequences of all specimens of *Sinodina* spp., but two ones of *Sinodinagregoriana*, have been obtained. No intraspecific variation in 16S of *Sinodinaashima* sp. nov. was detected, and the intraspecific p-distance of *Sinodinagregoriana* was 0.45%. The interspecific p-distances between *Sinodinaashima* sp. nov. and *Sinodinagregoriana* were 4.55% and 4.32%, between *Sinodinaashima* sp. nov. and *Sinodina* sp. were 2.05% and 2.27%, and between *Sinodinagregoriana* and *Sinodina* sp. was 4.09%.

## ﻿Discussion

Some morphological characteristics of *Sinodina* seem to be plesiomorphic. Its simple lamellar podobranch is the same as that of *Caridina* during the metamorphosis from the zoea to the first post-larval stage, without further development (Liang 2004). The distention and spininess on the distal ventral margin of the propodus of the male third and fourth pereiopod also appear in the basal genus *Paratya* whose pereiopods still possess exopods (Liang and Cai 1999; Liang 2004). Therefore, *Sinodina* is considered to be a relatively basal genus. In a previous phylogeny, *Sinodina* was detected as a sister group to all sampled taxa from China and Japan by three genes, including 16S, 18S and H3 (von Rintelen et al. 2012). However, our preliminary molecular analysis with low support values for the higher-level phylogenetic relationships does not reflect this relationship. To better clarify the taxonomic status and phylogenetic position of this genus, future studies should include more taxa and additional molecular data.

The new species with the simple and lamellar podobranch and its distribution is certainly a member of the genus *Sinodina*. This result is also supported by the phylogenetic analysis, in which *Sinodinaashima* sp. nov. is firmly clustered with the type species *Sinodinagregoriana*. As the first cave-dweller described in the genus, it can be easily distinguished from other species by its degraded body color and eyes. Besides, *Sinodinaashima* sp. nov. is similar to *S.heterodactyla* (Liang & Yan, 1985) and *S.banna* (Cai & Dai, 1999). They all show nearly no sexual dimorphism on the third and fourth pereiopod. The new species differs from the two species not only in the stygomorphic traits, but also in the extremely elongated and upturned rostrum, obviously beyond the end of scaphocerite (vs. *S.heterodactyla* reaching the end of scaphocerite and *S.banna* only reaching the end of the first segment of the antennular peduncle); the rostrum with 8–14 teeth ventrally (vs. 5–9 in *S.heterodactyla* and no ventral tooth in *S.banna*); the dactylus of male third pereiopod with 4–6 spines (vs. 7 in *S.heterodactyla* and 8–10 in *S.banna*); the body length 23–40 mm (vs. 23–32 mm in *S.heterodactyla* and 14–17 mm in *S.banna*).

Cave organisms in the karst region of south China have a long evolutionary history and high diversity (Li et al. 2022). In the past two decades, the knowledge of the subterranean fauna of China has rapidly increased, making this region a newly emerged world-class diversity hotspot (Deharveng and Bedos 2018). Our research adds a new generic-level taxon to the stygofauna of China. The first subterranean shrimp species from China was described in 1981, *Typhlocaridinalanceifrons* Liang & Yan, 1981, and 13 species have been published in the last century. Entering the 21^st^ century, there has been a significant increase in the rate of the discovery of subterranean atyids, with 15 species reported, 11 of which have been published in the last five years. It is believed that as the investigation goes further, more new species and new high-level taxa will be discovered.

## Supplementary Material

XML Treatment for Sinodina ashima

## ﻿Additional information

### Author ORCIDs

Xuankong Jiang https://orcid.org/0000-0003-3506-5894

Jiajun Zhou https://orcid.org/0000-0003-1038-1540

Jianguo Wang https://orcid.org/0009-0000-4675-4615

Wenlong Chen https://orcid.org/0009-0002-1170-3847

Huiming Chen https://orcid.org/0000-0002-2449-3036

### Data availability

All of the data that support the findings of this study are available in the main text.
